# Persistent hydrocephalus following posterior fossa tuberculoma removal in pediatrics: A case report from a referral center in Indonesia

**DOI:** 10.1016/j.ijscr.2024.110224

**Published:** 2024-08-29

**Authors:** Vega Pangaribuan, Muhammad Arifin Parenrengi, Wihasto Suryaningtyas

**Affiliations:** Department of Neurosurgery, Universitas Airlangga – Dr. Soetomo General Academic Hospital, Surabaya, East Java, Indonesia

**Keywords:** Tuberculosis, Pediatrics, Hydrocephalus, Tuberculomas, CSF diversion, Posterior fossa, Case report

## Abstract

**Introduction:**

The threat posed by tuberculosis persists in developing countries. Individuals under the age of five were more likely to develop central nervous system (CNS) tuberculosis. CNS Tuberculoma of the posterior fossa has rarely been reported, and its consequences are more devastating due to the limited space of the posterior fossa.

**Case presentation:**

A 4-year old male was referred to our academic general hospital with main complaint of decreased consciousness for the last 3 days. The patient has experienced a low-grade fever, cough, and an enlarging neck tumor for two months. Any contact with confirmed tuberculosis patients was denied by the family. A suspected cerebellar abscess and obstructive hydrocephalus led to the patient's referral. Urgent evacuation of the posterior fossa mass was conducted, revealing a histopathological diagnosis of tuberculoma. After the procedure, the patient experienced seizures and no improvement of GCS. A head CT scan evaluation revealed a communicating hydrocephalus. A ventriculoperitoneal shunt is done, resulting in improvement of the patient's consciousness after CSF diversion.

**Discussion:**

The haematogenous spread of Mycobacterium, which causes granulomatous foci in the brain, is the cause of CNS tuberculoma. The neuroradiological characteristic of tuberculomas may mimic several conditions. Thorough history-taking and physical examination may lead to a focused differential diagnosis of the patient. Evacuated posterior fossa tuberculoma usually leads to resolved obstructing hydrocephalus. A persistent hydrocephalus leads to the possibility of communicating hydrocephalus due to tuberculous meningoencephalitis. Close monitoring following excision of posterior fossa tuberculoma may help identify persistent hydrocephalus early on.

**Conclusion:**

CNS tuberculoma should remain a differential diagnosis of ring-enhancing posterior fossa mass, especially in pediatrics. This condition may present concomitantly with tuberculous meningoencephalitis, and it may be presented as a persistent hydrocephalus following the surgical removal of the lesion.

## Highlights

• Diagnosing CNS Tuberculoma is a challenge in pediatrics, since the clinical presentation may be atypical and the radiologic presentation may mimic other conditions.
• CNS Tuberculoma of the posterior fossa that leads to obstructive hydrocephalus should be evacuated as soon as possible due to the limited space of the posterior fossa.
• Removal of the obstructing mass should lead to resolve hydrocephalus; however, persistence of hydrocephalus should prompt clinician for the possibility of tuberculous meningoencephalitis.

## 1 Introduction

Tuberculosis infection is still prevalent in developing countries. As of 2021, Indonesia has the second-highest tuberculosis burden in the world, with an incidence rate of 354 per 100,000 people [1]. Children under the age of five and immunocompetent individuals are more susceptible to CNS tuberculosis [2].

The diagnosis of CNS tuberculoma remains a challenge since its radiologic presentation can mimic malignancy, parasitic infections, pyogenic abscesses, and lymphomas [3]. The clinical presentation itself may not be specific, and the appearance of pulmonary tuberculosis is not specific in pediatric patient, adding difficulties in diagnosing CNS tuberculoma in pediatric patients [4].

To aid in the diagnosis of tuberculosis, a complete history taking, laboratory examination, pulmonary x-ray, CT scan with contrast, mantoux test, CSF analysis, Xpert MTB/RID test, and specimen culture are encouraged to be done [5,6].

The presentation of CNS tuberculoma depends on the site of the mass. The patient may present with headache, nausea, diplopia, ataxia, and focal neurologic deficits [5]. Posterior fossa tuberculomas can also be accompanied by obstructive hydrocephalus due to close proximity to fourth ventricle and cerebral aqueduct [3,7].

Persistence of hydrocephalus following surgical removal of posterior fossa tuberculoma may occur. Several studies have reported the use of CSF diversion prior or during the surgical excision of this lesion [8,9]. Because of the potential for tuberculous meningoencephalitis-related hydrocephalus in this condition, CSF diversion should be done promptly.

This case report has been reported according to the SCARE (Surgical Case Report) Guidelines [10].

## 2 Case presentation

A 4-year old male was referred to our academic general hospital with main complaint of progressive loss of consciousness. Initially the patient had persistent fatigue 1 week prior. He also had enlarging neck lump, low grade fever, and mild cough for the past two months. The parents denied any contact with tuberculosis confirmed individuals, and had no family member with chronic cough. The patient also had vomiting 1 week prior. There's no weight loss, history of ear infection, or dental pain.

**Fig. 1:**
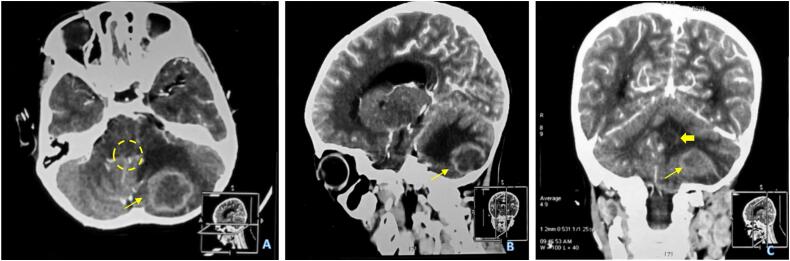
Head CT Scan with contrast revealing a multiple intra-axial mass of the posterior fossa. Axial cut of the head CT Scan (A), revealing a ring enhancing mass of the left cerebellum (thin arrow), causing mass effect and obliterating the fourth ventricle (dashed circle). Sagittal cut of the CT Scan (B). There's another intra-axial mass near the fourth ventricle causing distortion of the fourth ventricle (thick arrow). Non-contrast CT Scan revealed no contrast enhancement of the leptomeninges.

A head CT scan was done in the referring hospital, revealing multiple ring-enhancing masses of the cerebellum, with the largest mass sizing 3.3 cm × 3.0 cm × 2.2 cm (volume ± 12 cm^3^). There is enlargement of the lateral and third ventricles and a lack of leptomeningeal enhancement (Fig. 1). He was referred to our hospital with suspicion of a pyogenic brain abscess of the posterior fossa.

The physical examination revealed vital signs: heart rate of 131 bpm, a respiratory rate of 28 times per minute, temperature of 38.2C, and oxygen saturation of 98 %. There's dyspnea and subcostal retraction upon examination. The patient had Glasgow coma scale of 10 (opening eyes with verbal command, incomprehensible sounds, and able to localize pain). The patient had no positive meningeal signs, left occulomotor nerve palsy, and horizontal nystagmus. There's weakness of the left extremities and positive pathological reflexes.

**Fig. 2:**
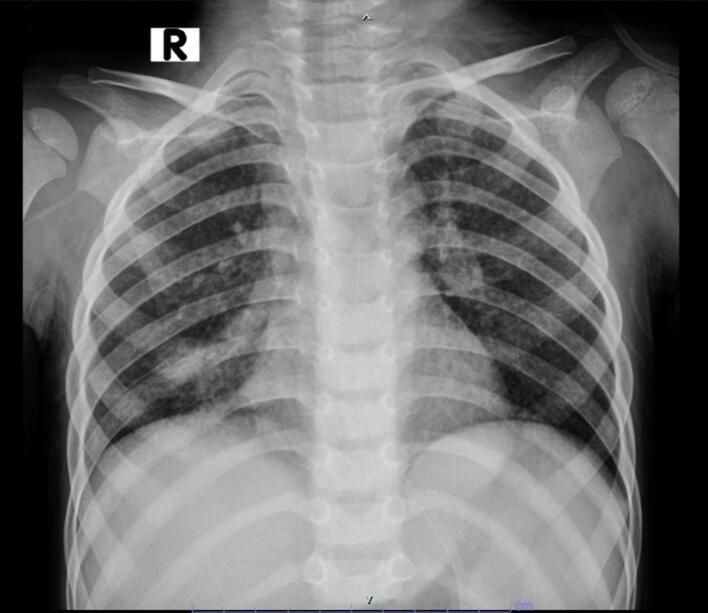
Chest X-ray revealed a right parahilar infiltrates.

An opthalmologic consult was done, revealing an early papiledema of both eyes (Frisen scale grade 1). A chest x-ray was done and revealed infiltrates in the right parahilar area (Fig. 2).

**Fig. 3:**
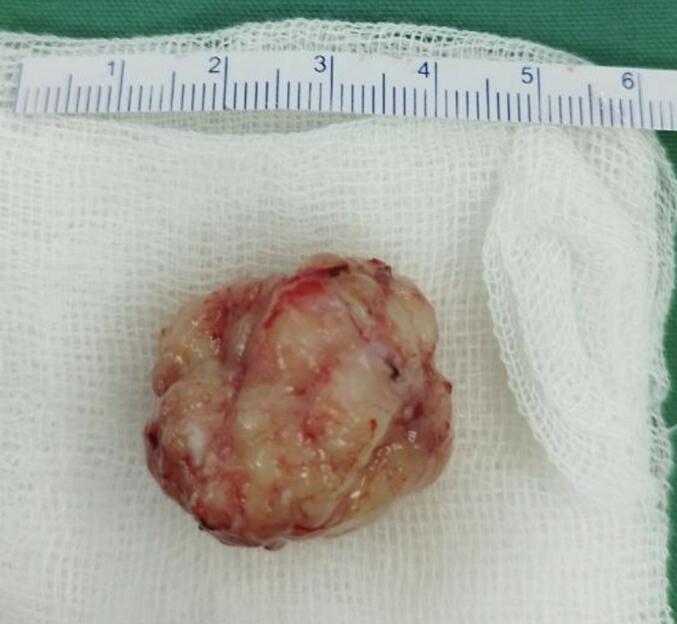
A greyish solid mass with soft consistency, with size 3 cm × 2.8 cm × 3.1 cm, consistent with macroscopic appearance of the tuberculoma.

An urgent removal of the posterior fossa mass was done. A left paramidline incision is made on top of the scannogram of the lesion. Upon incison of the duramater, the mass appears solid and soft in consistency, thus excluding the possibility of pyogenic abscess or cystic mass. The lesion was separated from the surrounding arachnoidmater using a dissector. It was removed completely, revealing a solid greyish mass sized 3 cm × 2.8 cm × 3.1 cm (Fig. 3). As the obstructive mass had been removed, the surgical site was closed layer by layer. With his intubation in place, the patient was admitted to the intensive care unit.

**Fig. 4:**
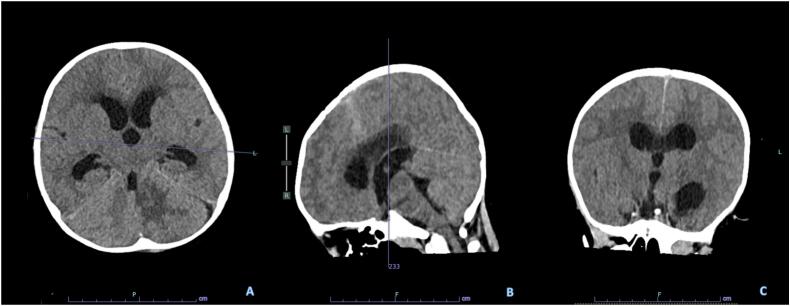
Head CT Scan with without contrast, revealing a complete removal of the tuberculoma mass, there's no obstruction of the ventricular system. As there are dilatation of all the cerebral ventricles, suspicion of tuberculous meningoencephalitis was raised.

Upon observation, the patient had no improvement of GCS, and there was a tonic-clonic seizure for 5 min. An evaluation funduscopy was done, revealing a bilateral papiledema frisen scale grade one. A non-contrast head CT scan evaluation was done in the same day, revealing a communicating hydrocephalus (Fig. 4). The patient was immediately sent to the emergency operating room for ventriculoperitoneal shunt placement at the right kocher point.

**Fig. 5:**
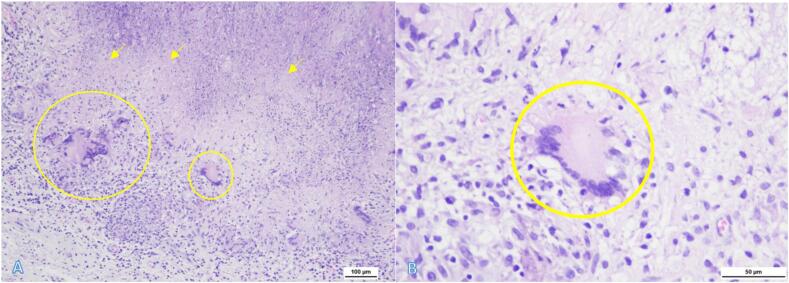
Hematoxylin eosin staining of the posterior fossa tissue reveals a large area of necrosis (arrows), at the edge of which there is a cluster of inflammatory cells of histiocytes in the form of epitheloid forming a granuloma (circles), surrounded by inflammatory cells of lymphocytes, neutrophils and plasma cells (100× hpf) (A). Giant langhans cell with caseous necrotic is visible at the edge of the granuloma (400× hpf) (circle) (B). There are no signs of malignancy.

The genXpert of the tissue was positive for *Mycobacterium tuberculosis* and sensitive for rifampicin. Interestingly, the genXpert of the gastric lavage result is negative. The pathological anatomy of the tumor tissue is consistent with tuberculoma, revealing a large area of necrosis with multiple multinucleated giant cells (Fig. 5). CSF examination revealed clear CSF, with glucose content of 91.2 g/dL and protein content of 9.74 g/dL.

Tuberculosis drug therapy was immediately started with an intensive tuberculous regimen consisting of rifampicin, isoniazide, pyrazinamide, and ethambutol. The seizures were controlled using phenytoin and phenobarbital. The patient's consciousness was progressively getting better, and he was discharged after 3 weeks of admission with GCS of E4V2M5. The parents were educated to continue the tuberculosis management for one year and routine visits to the neurosurgery and pediatrics outpatient clinic.

## 3 Discussion

Tuberculosis is a prevalent disease; in certain population its consequences can be devastating. Children younger than 5 years old are prone to tuberculous CNS, and the manifestations of the pulmonary TB may not be typical [4]. This poses a diagnostic challenge and should prompt clinicians to conduct extensive history taking, physical examination, laboratory, and radiological examination [11].

Intracranial tuberculoma appearance can mimics many disease, such as cerebral parasitic infestations, pyogenic abscess, and brain tumours. It can appear solitary or multiple and its components can be solid or cystic [12]. In contrast enhanced head CT Scan, tuberculoma can appear with hypo or hyperdense mass with ring enhancement. The appearance of a caseous center surrounded by ring-like enhancement is the most common presentation of tuberculoma in head CT scan [13]. However, it may appear as an iso- or mixed-density lesion, depending on the consistency of tuberculoma [11,14].

In this case report, the patient presented with multiple intra-axial masses of the cerebellum. One lesion is located superficially on the left cerebellum, and one smaller lesion is located near the fourth ventricle, both causing obstructive hydrocephalus. Following the surgical removal of the tuberculoma, there's no obstruction of the cerebral ventricles; however, the hydrocephalus persisted in the non-contrast head CT scan evaluation.

Persistence of hydrocephalus following posterior fossa lesions have been well described in pediatric tumor cases [15,16]. There has been no recommendation or guidelines regarding the use of CSF diversion in posterior fossa tuberculomas. The persistence of hydrocephalus following the posterior fossa tuberculoma removal may have correlation with the existence of tuberculous meningoencephalitis [17]. The use of CSF diversion, including external ventricular diversion (EVD), endoscopic third ventriculostomy (ETV), and ventriculoperitoneal shunt, has been documented in several cases of posterior fossa tuberculomas [[18], [19], [20], [21]]. A report by Escobedo-Meléndez described a posterior fossa tuberculoma case in which the patient presented with hydrocephalus; thus, the patient immediately underwent ventriculoperitoneal shunt placement. In this case, the MRI was conducted several days later [19]. A similar case was also described by Simsek et al., where the patient presented initially with hydrocephalus and meningoencephalitis; thus, the patient received a ventriculoperitoneal shunt prior to the identification of posterior fossa tuberculomas [20].

The management of the intracranial tuberculoma depends on the location of the lesion, mass effects, and the need to obtain a pathological anatomy sample [21]. Surgical removal of the lesion is advised for patients who exhibit signs of high intracranial pressure, hydrocephalus due to obstruction from tuberculoma's mass effects, or superficial lesions that do not involve the eloquent area [5].

Tuberculoma can present with meningoencephalitis; it may occur due to haematogenous spread or rupture of granulomatous foci into the subarachnoid space. Tuberculous meningoencephalitis diagnosis is quite challenging; the CSF analysis usually presents with high CSF protein (100–500 mg/dL), lymphocyte predominant pleocytosis (100–500 cells/μl), and low glucose (<45 mg/dL) [17]. In our case, the CSF analysis result was negative for tuberculous meningitis. However, a negative ventricle CSF sample should not exclude a tuberculous meningitis diagnosis; since Mycobacterium bacteriological yields may not appear in ventricular CSF [22].

The patient's immediate family feels that the doctor's recommendations were all for the patient's best interests and is always grateful for them during treatment. In order to treat the patient's condition, the patient's family trusts the doctor to do whatever is necessary. The patient's family expresses their appreciation for the doctor's care as well as the fact that the patient's condition is gradually getting better.

## 4 Conclusion

As tuberculosis is still prevalent in Indonesia, children presenting with ring-enhancing intra-axial mass should always be suspected of having tuberculomas. Obstructive hydrocephalus can result from mass effects of posterior fossa tuberculoma. The persistence of hydrocephalus following the lesion's surgical removal may occur in patients with concurrent tuberculous meningoencephalitis. Vigilant clinical observation and early radiological evaluation can lead to early diagnosis of this condition. CSF diversion can be done, however it should be personalised for each patient's individual cases.

## Consent

Written informed consent was obtained from the patient for publication of this case report and accompanying images. A copy of the written consent is available for review by the Editor-in-Chief of this journal on request.

## Provenance and peer reviews

Not commissioned, externally peer-reviewed.

## Ethical approval

Ethical approval for this study was waived by the Ethics Committee because the anonymity of patient was assured and no more than two subjects were included in this study.

## Funding

None.

## Author contribution

Vega Pangaribuan – manuscript writing, data collection, revising, and reviewing the final version of the article

Muhammad Arifin Parenrengi –study conception, patient contribution, article revising, figures creation, study oversight

Wihasto Suryaningtyas – data collection, revising, and reviewing the final version of the article

## Guarantor

MP accepts full responsibility for this review manuscript.

## Research registration number

Not applicable.

## Conflict of interest statement

The authors report there are no competing interests to declare.
